# Supplementary material for: Identification of Freezing-Responsive microRNAs and Their Targets in Chinese Jujube by Small RNA and Degradome Sequencing
Source: Int J Mol Sci. 2026 Sep 4;27(17):7912. doi: 10.3390/ijms27177912 (PMC13566890; doi:10.3390/ijms27177912)
Supplement: Supplementary file 1 [file ijms-27-07912-s001.zip › Supplementary Material S2/novel_miR_101.pdf]

Provisional ID	:	novel_miR_101
Score total	:	7166.8
Score for star read(s)	:	3.9
Score for read counts	:	7158.1
Score for mfe	:	3.3
Score for randfold	:	1.6
Score for cons. seed	:	
Total read count	:	14052
Mature read count	:	9107
Loop read count	:	29
Star read count	:	4916

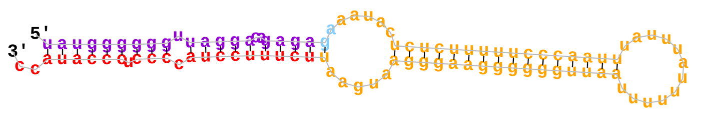

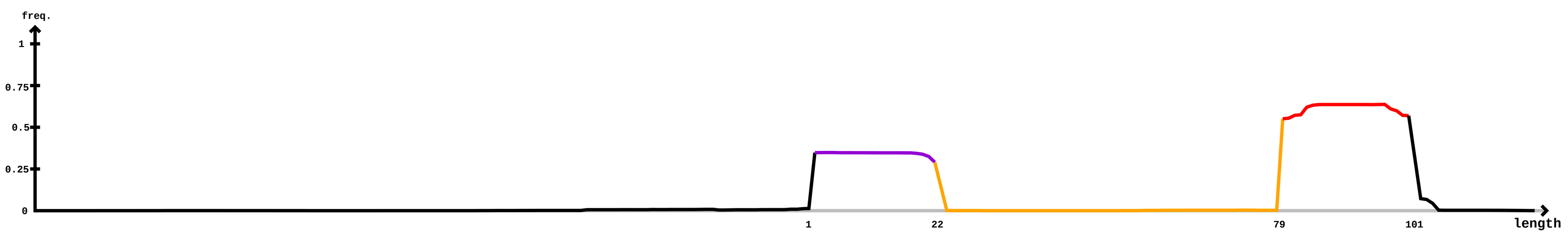

Star Mature

```
5'- ccuuuuuuucaccaaaaucuagacucaauuaauuucucuuuucccuuccuccaccaucugguguauuucuuuucucacuaaaaggauggaagagggaaaggcuuaagagugugggugggaagugauagguaugggggguuaggacagagagaaauacucucuuuucccaauuuauuuauuuuuuaauugggggaagggaaugaauucuuuccuaccccucccauaccauucauuuccauccuuuccuuc -3'  obs
    ccuuuuuuucaccaaaaucuagacucaauuaauuucucuuuucccuuccuccaccaucugguguauuucuuuucucacuaaaaggauggaagagggaaaggcuuaagagugugggugggaagugauagguaugggggguuaggacagagagaaauacucucuuuucccaauuuauuuauuuuuuaauugggggaagggaaugaauucuuuccuaccccucccauaccauucauuuccauccuuuccuuc       exp
    ..................................((((((((((..((((((...(((((...........)))))...)).)))).))))))))))...(((.(...(((((((((((..(((((((((((.(((((..(((((.....(((((((((((((((...........))))))))))))))))).....))))))))))).))).))))))))..))))))))))))...)))).
```

read	reads	mm	sample
caccaaaaucuagacucaauu	1	0	LT-1
ccaaaaucuagacucaauuaauuucucu	1	0	JCK-2
aaucuagacucaauuaauuucuc	1	0	LT-3
uagacucaauuaauuucucuuuuc	1	0	JCK-1
agacucaauuaauuucucuuuucc	1	0	JCK-1
agacucaauuaauuucucuuuucc	1	0	JCK-3
agacucaauuaauuucucuuuucc	1	0	JCK-2
ucugguguauuucuuuucuc	2	0	JCK-3
ucucacuaaaaggauggaagagggaa	2	0	LT-1
ucucacuaaaaggauggaagagggaaagg	1	0	JCK-1
ucucacuaaaaggauggaagagggaaagg	1	0	JCK-3
cucacuaaaaggauggaagagggaaagg	1	0	JCK-3
cacuaaaaggauggaagagg	1	0	LT-3
cacuaaaaggauggaagagggaaagg	1	0	JCK-2
cacuaaaaggauggaagagggaaaggA	2	1	JCK-3
cacuaaaaggauggaagagggaaaggA	2	1	JCK-2
aaaaggauggaagagggaaagg	4	0	LCK-3
aaaaggauggaagagggaaagg	1	0	LT-3
aaaggauggaagagggaaagg	1	0	LT-3
aggauggaagagggaaagg	3	0	LT-2
agagggaaaggcuuaagagu	5	0	LT-2
agagggaaaggcuuaagagug	1	0	LT-3
agagggaaaggcuuaagagugu	36	0	LCK-2
agagggaaaggcuuaagagugu	21	0	LCK-1
gagggaaaggcuuaagagug	1	0	JCK-2
Cgggaaaggcuuaagagugugggg	1	1	LT-1
aaaggcuuaagagugugggugg	1	0	LT-3
aaaggcuuaagagugugggugggaagu	2	0	JCK-1
aggcuuaagagugugggug	1	0	LT-1
cuuaagagugugggugggaaguga	2	0	JT-2
cuuaagagugugggugggaaguga	2	0	JT-3
cuuaagagugugggugggaagugauag	2	0	JCK-3
cuuaagagugugggugggaagugauag	2	0	JCK-2

Star **Mature**

ccuuuuuuucaccaaaaucuagacucaauuaauuucucuuuucccuuccuccaccaucugguguauuucuuuucucacuaaaaggauggaagagggaaaggcuuaagaguguggguggaagugauagg**uaugggggguuaggacagaga**gaaauacucucuuuucccaauuuauuuauuuuuuaauuggggggaagggaaugaau**ucuuuccuaccccucccauacc**auucauuuccauccuuuccuuc

cuuaagaguguggguggaagugauagg	1	0	LT-2
cuuaagaguguggguggaagugauagg	2	0	LT-1
cuuaagaguguggguggaagugauagg	1	0	JCK-3
cuuaagaguguggguggaagugauagg	5	0	JCK-2
cuuaagaguguggguggaagugauagg	4	0	JT-2
cuuaagaguguggguggaagugauagg	3	0	JT-3
aagaguguggguggaagugauagg	4	0	JT-3
aagaguguggguggaagugauagg	2	0	JT-2
guguggguggaagugauagg	1	0	JCK-3
guguggguggaagugauagg	1	0	JCK-1
uguggguggaagugauaggu	10	0	LCK-3
gugggugggaagugauagguau	1	0	LT-1
guggguggaagugauagguaug	8	0	LCK-3
uggguggaagugauagguaugg	2	0	JCK-3
gguggaagugauagguauggg	1	0	LT-3
guggaagugauagguaug	5	0	LCK-3
guggaagugauagguauggg	1	0	LT-1
guggaagugauagguaugggg	2	0	JCK-2
guggaagugauagguaugggg	1	0	JCK-3
uggaagugauagguaugg	1	0	LT-3
uggaagugauagguaugggg	1	0	JCK-3
uggaagugauagguaugggg	2	0	JCK-2
uggaagugauagguaugggggg	6	0	LCK-3
agugauagguaugggggguu	3	0	LT-3
agugauagguaugggggguuaggaca	7	0	LCK-3
agugauagguaugggggguuaggacagaga	2	0	LCK-3
ugauagguaugggggguuaggaca	3	0	LT-3
auagguaugggggguuaggacaga	1	0	JCK-2
auagguaugggggguuaggacaga	1	0	JCK-1
uagguaugggggguuagga	4	0	LT-3
uagguaugggggguuagga	1	0	JCK-1
uagguaugggggguuaggac	2	0	JCK-2
uagguaugggggguuaggacU	1	1	LT-3
uagguaugggggguuaggacC	1	1	LT-3
uagguaugggggguuaggaca	1	0	LT-3
uagguaugggggguuaggaca	4	0	LCK-3
uagguauggggggAuuaggaca	1	1	LT-1
uagguaugggggguuaggaca	6	0	JCK-2
uagguaugggggguuaggaca	6	0	JCK-3
uagguaugggggguuaggacC	2	1	JCK-3
uagguaugggggguuaggaca	1	0	JCK-1
uagguaugggggguuaggaca	3	0	LT-1
uagguaugggggguuaggaca	1	0	JT-2
uagguaugggggguuaggaca	3	0	JT-3
uagguaugggggguuaggacaga	1	0	JCK-3
gguaugggggguuaggacag	1	0	JCK-3
gguaugggggguuaggacaga	4	0	LT-1
gguaugggggguuaggacaga	3	0	LT-3
gguaugggggguuaggacaga	4	0	JCK-3
gguaugggggguuaggacaga	12	0	JT-1
gguaugggggguuaggacaga	9	0	JCK-1
gguaugggggguuaggacaga	9	0	LT-2
gguaugggggguuaggacaga	1	0	LCK-3
gguaugggggguuaggacaga	3	0	JCK-2
guaugggggguuaggacaga	1	0	LT-3
guaugggggguuaggacaga	6	0	LCK-3
guaugggggguuaggacaga	2	0	LT-1
guaugggggguuaggacagag	1	0	JCK-3
Cuaugggggguuaggacagaga	2	1	JCK-2
Cuaugggggguuaggacagaga	2	1	JCK-1
Cuaugggggguuaggacagaga	1	1	LT-3
Cuaugggggguuaggacagaga	7	1	JT-1
uaugggggguuaggacag	10	0	LT-3
uaugggggguuaggacag	9	0	JCK-1
uaugggggguuaggacag	8	0	LT-2
uaugggggguuaggacag	8	0	LT-1
uaugggggguuaggacag	18	0	JCK-3
uaugggggguuaggacag	14	0	JCK-2
uaugggggguuaggacag	10	0	LCK-3
uaugggggguuCggacag	1	1	JCK-2

Star (uaugggggguuaggacagaga) — **Mature** (ucuuuccuaccccucccauacc)

ccuuuuuucaccaaaaucuagacucaauuaauuucucuuuucccuuccuccaccaucugguguauuucuuuucucacuaaaaggauggaagagggaaaggcuuaagagugugggguggaagugauagg**uaugggggguuaggacagaga**gaaauacucucuuuuucccaauuuauuuauuuuuuaauuggggggaagggaaugaau**ucuuuccuaccccucccauacc**auucauuuccauccuuuccuuc

...uaugggggguuaggacaga...	37	0	LCK-3
...uaugggggguuaggacaga...	5	0	JT-3
...uNugggggguuaggacaga...	1	1	JT-2
...uCugggggguuaggacaga...	1	1	JCK-1
...uaugggggguuaggacaga...	14	0	LT-3
...uaugggggguuaggacaga...	11	0	JT-2
...uaugggggguuaggacaga...	11	0	JCK-2
...uaugggggguuaggacagU...	1	1	LT-1
...uaugggggguuaggacaga...	12	0	JCK-3
...uaugggggguuaggacagU...	1	1	JCK-2
...uaugggggguuaggacaga...	19	0	LT-2
...uaugggggguuaggacaga...	11	0	JT-1
...uaugggggguuaggacaga...	8	0	LT-1
...uaugggggguuaggacagag...	7	0	JT-2
...uaugggggguuaggacagag...	20	0	JT-3
...uCugggggguuaggacagag...	1	1	JT-3
...uaugggggguuaggacagag...	91	0	JCK-2
...uaugggggguuaggacagag...	34	0	LT-1
...uaugggggguuaggacagag...	43	0	LT-3
...uCugggggguuaggacagag...	3	1	JCK-2
...uaugggggguuaggacagag...	16	0	LCK-3
...uaugggggguuaggacagag...	60	0	JCK-1
...uaugggggguuaggacagag...	62	0	JCK-3
...uaugggggguuaggacagaU...	1	1	LT-2
...uaugggggguuCggacagag...	1	1	JCK-2
...uaugggggguuaggacagag...	115	0	JT-1
...uaugggggguuaggacagaN...	1	1	JCK-1
...uaugggggguuNggacagag...	1	1	JCK-1
...uaugggggguuaggacagag...	35	0	LT-2
...uauAggggguuaggacagaga...	1	1	JCK-1
...uaugggggguuaggacagagU...	3	1	LT-3
...uaugggggguuaggacagaga...	206	0	LT-1
...uNugggggguuaggacagaga...	1	1	JCK-2
...uauAggggguuaggacagaga...	1	1	JCK-2
...uaugggggguuaggacCgaga...	1	1	JCK-2
...uaugggggguuCggacagaga...	1	1	LCK-3
...uaugggggguuaggacagaga...	192	0	JT-2
...Caugggggguuaggacagaga...	1	1	LT-2
...uaugggggguuaggacagaNa...	1	1	JCK-1
...uaugggggguuaggacagCga...	1	1	LT-2
...uCugggggguuaggacagaga...	1	1	JCK-3
...uaugggggguuaggacagaga...	555	0	JCK-3
...uaCgggggguuaggacagaga...	1	1	JT-3
...uaugggggguuCggacagaga...	1	1	LCK-2
...uaugggggguuaggCcagaga...	1	1	JCK-1
...uaugggggguuaggacagaga...	242	0	LT-2
...uaugggggguuaggCcagaga...	2	1	JCK-2
...uaugggggguuaggCcagaga...	1	1	JCK-3
...uaugggggguuaggacagaga...	727	0	JCK-2
...uaugggggguuCggacagaga...	1	1	JCK-3
...uaugggggguuaggacagagU...	1	1	JCK-1
...uaNgggggguuaggacagaga...	1	1	LCK-2
...uaugggUgguuaggacagaga...	1	1	JT-3
...uaugggggguuagAacagaga...	1	1	LCK-3
...uaugggggguuaggacagaga...	113	0	LCK-1
...uauAggggguuaggacagaga...	1	1	JCK-3
...uCugggggguuaggacagaga...	2	1	LCK-3
...uaugggggguuaggacagagC...	2	1	JCK-1
...uaugggggguuagUacagaga...	1	1	LCK-2
...uCugggggguuaggacagaga...	1	1	JT-3
...uCugggggguuaggacagaga...	3	1	JT-1
...uaugggggguuaggacagaga...	589	0	JT-1
...uaugggggguuNggacagaga...	1	1	JCK-1
...uaugggggguuaggacagagC...	2	1	JCK-3
...uaugggggguuaggacagaga...	289	0	LT-3
...uauggggggCuaggacagaga...	1	1	JT-2
...uaugggggguuaggacagagU...	1	1	LT-1
...uaugggggguuaggCcagaga...	1	1	LT-3
...uaugggggguuagNacagaga...	2	1	LCK-1
...uaugggggguuaggacagaga...	88	0	LCK-2

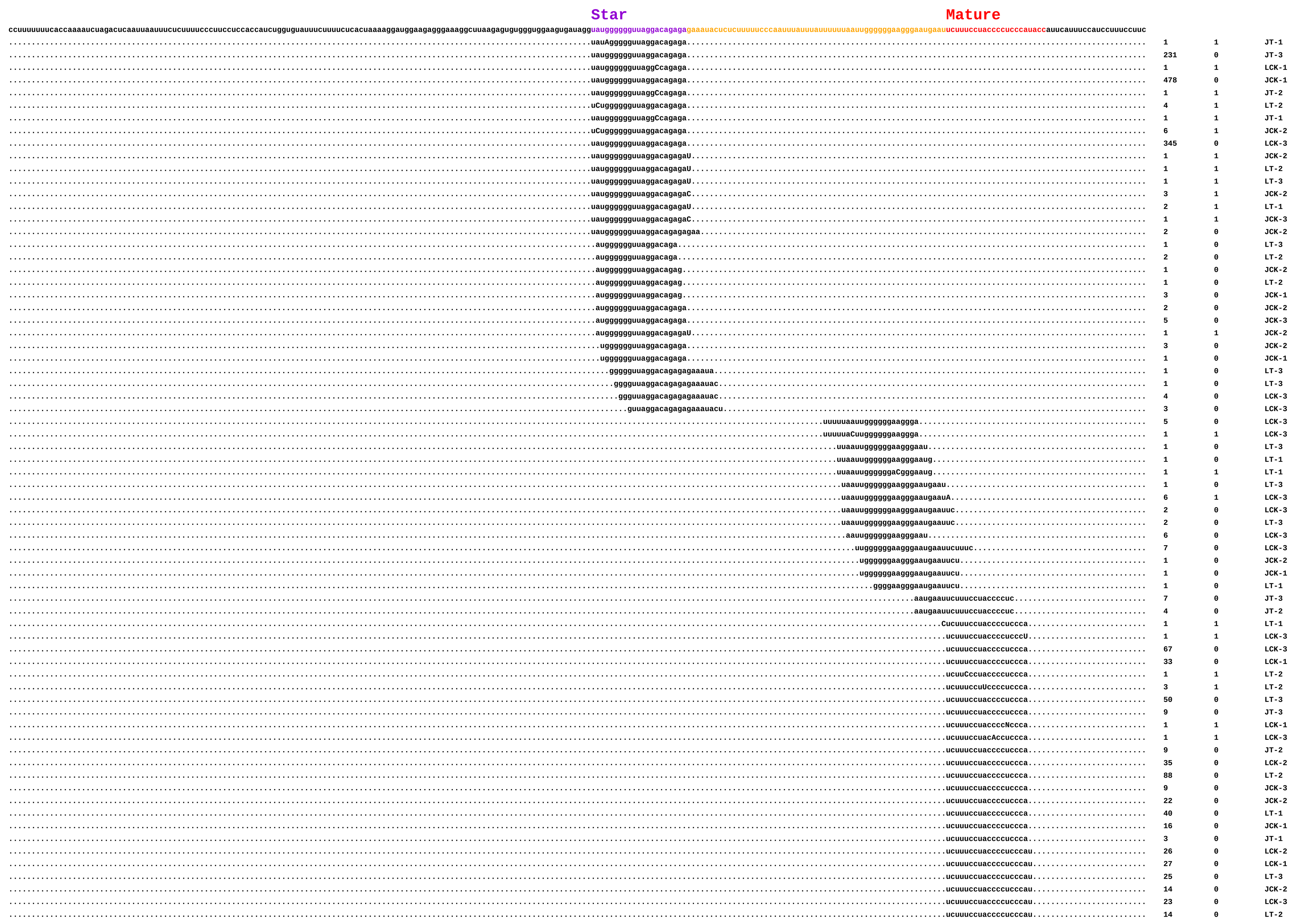
Star
Mature

Star / Mature

ccuuuuuuucaccaaaaucuagacucaauuaauuucucuuuucccuuccuccaccaucugguguauuucuuuucucacuaaaaggauggaagagggaaaggcuuaagagugugggugguggaagugauagguaugggggguuaggacagagagaaauacucucuuuuucccaauuuauuuauuuuuuaauugggggaagggaaugaauucuuuccuaccccucccauaccauucauuuccauccuuuccuuc

ucuuuccuaccccuGccau	1	1	LCK-1
ucuuuccuaccccucccau	6	0	JCK-3
ucuuuccuaccccuAccau	1	1	LCK-3
ucuuuccuaccccucccau	12	0	JCK-1
ucuuuccuaccccucccau	17	0	LT-1
ucuuuccuaccccucccaua	59	0	JCK-2
uNuuuccuaccccucccaua	1	1	JCK-2
ucuuuccuaccccucccauU	2	1	LT-3
ucuuuccuaccccucccaua	42	0	LT-1
ucuuuccuaccccucccaua	35	0	LT-3
ucuuuccuaccccucccaua	53	0	LCK-3
ucuuuccuaccccucccaua	43	0	JCK-1
ucuuuccuaccccucccaua	41	0	LT-2
ucuuuccuaccccucccauC	2	1	LT-3
ucuuuccuaccccucccaua	57	0	JCK-3
ucuuuccuaccccucccauU	2	1	LT-1
ucuuuccuaccccucccCua	1	1	LT-3
ucuuuccuacUccucccaua	28	1	JT-1
ucuuuccuaccccucccaAa	1	1	JCK-3
ucuuuccuaccccucccauac	7	0	JCK-2
ucuuuccuaccccucccauac	8	0	LT-2
ucuuuccuaccccucccauac	5	0	JCK-3
ucuuuccuaccccucccauac	1	0	JCK-1
ucuuuccuaccccucccauac	3	0	LT-1
ucuuuccuaccccucccauaA	1	1	LT-1
ucuuuccuaccccucccauac	4	0	LT-3
ucuuuccuaccccucccCuacc	1	1	LT-2
ucuuuccuacUccucccauacc	87	1	LCK-1
ucuuuccuaccccucccauacc	226	0	LT-2
ucuuuccuaccccucccauacc	48	0	JT-1
ucuuuccuaccccucccauCcc	1	1	JCK-1
ucuuuccuaAcccucccauacc	2	1	JCK-1
ucuuuccuacUccucccauacc	14	1	JT-2
ucuCuccuaccccucccauacc	1	1	LT-3
ucuuuccuCccccucccauacc	1	1	LT-1
ucCuuccuaccccucccauacc	1	1	JCK-2
ucuuuccuaccNcucccauacc	3	1	JCK-1
ucuuuccuaccccucccauacc	26	0	LCK-1
ucuuuccuaccccucUcauacc	1	1	LCK-3
ucuuuccuaccccCcccauacc	1	1	JCK-2
ucuuuccuaccccucccauaUc	1	1	JCK-3
ucuuuccuacccAucccauacc	1	1	LCK-3
ucuGuccuaccccucccauacc	1	1	JCK-1
ucCuuccuaccccucccauacc	1	1	LT-2
ucuuuccuacUccucccauacc	80	1	LCK-2
ucuuuccuCccccucccauacc	3	1	JCK-3
Ccuuuccuaccccucccauacc	2	1	LT-2
ucuuuccuacGccucccauacc	2	1	JCK-1
ucuuuccuaccccucccauacc	18	0	LCK-2
ucuuuccuCccccucccauacc	2	1	LT-3
uNuuuccuaccccucccauacc	1	1	JCK-3
ucuuucUuaccccucccauacc	3	1	JCK-2
ucuuuccuaccccucccauaGc	1	1	JCK-1
ucuuuccuaccccucccauacc	1673	0	JCK-3
ucuuuccuaccccucccauacc	419	0	LT-3
ucuuuccuaccccucccauCcc	1	1	LT-2
ucCuuccuaccccucccauacc	1	1	JCK-3
ucuuuccuaccccucccauCcc	2	1	LT-3
ucuuuccuaccccucccCuacc	2	1	JCK-3
ucuuuccuaccccuUccauacc	1	1	JCK-3
ucuuCccuaccccucccauacc	1	1	JCK-3
ucuuuccuaccccucccauacc	1146	0	JCK-1
ucuuuccuaccccucccauacU	2	1	JCK-1
ucuuuUcuaccccucccauacc	4	1	JCK-2
ucuuuccuaccccucccauacc	434	0	LT-1
uNuuuccuaccccucccauacc	1	1	JCK-1
ucuuuccuaccccuccUauacc	1	1	JCK-2
ucuuuccuaccccucccauaUc	1	1	JCK-2
ucuuuccuacGccucccauacc	2	1	JCK-2
ucuuuUcuaccccucccauacc	1	1	JCK-1

```
                                                                                                                                Star                                                                        Mature
ccuuuuuucaccaaaaucuagacucaauuaauuucucuuuucccuuccuccaccaucugguguauuucuuuucucacuaaaaggauggaagagggaaaggcuuaagagugugggguggaagugauagguauggggguuaggacagagagaaauacucucuuuuucccaauuuauuuauuuuuuaauuggggggaagggaaugaauucuuuccuaccccucccauaccauucauuuccauccuuuccuuc
.............................................................................................................................................................................................................ucuuuccuacGccucccauacc......................   3      1    JCK-3
.............................................................................................................................................................................................................ucuuuccuaccccucccauacc......................   29     0    JT-2
.............................................................................................................................................................................................................ucuuuccuaccccucccauaGc......................   1      1    JCK-2
.............................................................................................................................................................................................................ucuuuccuacUccucccauacc......................   26     1    JT-3
.............................................................................................................................................................................................................ucuuuccuaccccuccUauacc......................   2      1    LT-3
.............................................................................................................................................................................................................uUuuuccuaccccucccauacc......................   3      1    JCK-2
.............................................................................................................................................................................................................Ccuuuccuaccccucccauacc......................   1      1    JCK-3
.............................................................................................................................................................................................................ucuuuccuaccccucccauacU......................   1      1    LT-1
.............................................................................................................................................................................................................ucuuuccuaccccucccauacA......................   1      1    JCK-2
.............................................................................................................................................................................................................ucuuuccuaccccucccauacc......................   2190   0    JCK-2
.............................................................................................................................................................................................................ucuuuccuaccccucccCuacc......................   6      1    JCK-2
.............................................................................................................................................................................................................ucuuuccuacUccucccauacc......................   114    1    JT-1
.............................................................................................................................................................................................................ucuuucUuaccccucccauacc......................   1      1    JCK-1
.............................................................................................................................................................................................................ucuuuccuacUccucccauacc......................   1      1    JCK-2
.............................................................................................................................................................................................................uNuuuccuaccccucccauacc......................   4      1    JCK-2
.............................................................................................................................................................................................................ucuuuccuaccccuUccauacc......................   4      1    JCK-1
.............................................................................................................................................................................................................Ccuuuccuaccccucccauacc......................   3      1    JCK-2
.............................................................................................................................................................................................................ucuuuccuaccccucccauCcc......................   1      1    LT-1
.............................................................................................................................................................................................................ucuuuccuaUcccucccauacc......................   3      1    LT-3
.............................................................................................................................................................................................................uUuuuccuaccccucccauacc......................   1      1    JCK-3
.............................................................................................................................................................................................................ucuuuccuaccccucccGuacc......................   1      1    JCK-1
.............................................................................................................................................................................................................ucuuuccuaccccucccauCcc......................   3      1    JCK-2
.............................................................................................................................................................................................................ucuuuccuCccccucccauacc......................   7      1    JCK-2
.............................................................................................................................................................................................................ucuuuccuaccccucccauacc......................   18     0    JT-3
.............................................................................................................................................................................................................ucuuuccuaccccucccauacc......................   214    0    LCK-3
.............................................................................................................................................................................................................ucuuuccuaccccucccauNcc......................   1      1    JCK-1
.............................................................................................................................................................................................................ucCuuccuaccccucccauacc......................   1      1    JCK-1
.............................................................................................................................................................................................................uUuuuccuaccccucccauacc......................   1      1    JCK-1
.............................................................................................................................................................................................................ucuuuccuaccccucccCuacc......................   4      1    JCK-1
.............................................................................................................................................................................................................ucuuuccuaccccucccauaccU.....................   11     1    LT-1
.............................................................................................................................................................................................................ucuuuccuaccccucccauacca.....................   1      0    LT-1
.............................................................................................................................................................................................................ucuuuccuaccccucccauaccU.....................   2      1    JCK-2
.............................................................................................................................................................................................................ucuuuccuaccccucccauacca.....................   1      0    JCK-2
.............................................................................................................................................................................................................ucuuuccuaccccucccauaccU.....................   5      1    LT-3
.............................................................................................................................................................................................................ucuuuccuaccccucccauaccU.....................   6      1    JCK-1
.............................................................................................................................................................................................................ucuuuccuaccccucccauacca.....................   2      0    LT-3
.............................................................................................................................................................................................................ucuuuccuaccccucccauacca.....................   1      0    JCK-1
.............................................................................................................................................................................................................ucuuuccuaccccucccauaccU.....................   2      1    JCK-3
.............................................................................................................................................................................................................ucuuuccuaccccucccauaccUu....................   2      1    LT-2
.............................................................................................................................................................................................................ucuuuccuaccccucccauaccUu....................   6      1    LCK-3
.............................................................................................................................................................................................................ucuuuccuaccccucccauaccaA....................   3      1    LT-2
.............................................................................................................................................................................................................ucuuuccuaccccucccauaccUuu...................   1      1    LT-3
.............................................................................................................................................................................................................ucuuuccuaccccucccauaccUuu...................   3      1    LT-2
.............................................................................................................................................................................................................ucuuuccuaccccucccauaccauuG..................   1      1    LT-1
.............................................................................................................................................................................................................ucuuuccuaccccucccauaccaCuc..................   1      1    JCK-2
..............................................................................................................................................................................................................cuuuccuaccccucccau.........................   1      0    JCK-3
..............................................................................................................................................................................................................cuuuccuaccccucccau.........................   2      0    LCK-3
..............................................................................................................................................................................................................cuuuccuaccccucccaua........................   1      0    JCK-1
..............................................................................................................................................................................................................cuuuccuaccccucccaua........................   1      0    JCK-3
..............................................................................................................................................................................................................cuuuccuaccccucccaua........................   4      0    LCK-3
..............................................................................................................................................................................................................cuuuccuaccccucccauacc......................   6      0    LT-3
..............................................................................................................................................................................................................cuuuccuaccccucccauacc......................   16     0    JCK-3
..............................................................................................................................................................................................................cuuuccuaccccucccauacc......................   11     0    JCK-1
..............................................................................................................................................................................................................cuuuccuaccccucccauacc......................   11     0    JCK-2
..............................................................................................................................................................................................................cuuuccuaccccucccauaccauuU..................   2      1    JCK-3
..............................................................................................................................................................................................................cuuuccuaccccucccauaccauuU..................   2      1    JCK-2
...............................................................................................................................................................................................................uuuccuaccccucccaua........................   1      0    LT-2
...............................................................................................................................................................................................................uuuccuaccccucccauU........................   1      1    LT-3
...............................................................................................................................................................................................................uuuccuaccccucccaua........................   4      0    LCK-3
...............................................................................................................................................................................................................uuuccuaccccucccaua........................   1      0    JCK-2
...............................................................................................................................................................................................................uuuccuaccccucccaua........................   5      0    JCK-3
...............................................................................................................................................................................................................uuuccuaccccucccaua........................   1      0    LT-1
...............................................................................................................................................................................................................uuuccuaccccucccaua........................   2      0    LT-3
...............................................................................................................................................................................................................uuuccuaccccucccauacU......................   1      1    LT-1
...............................................................................................................................................................................................................Cuuccuaccccucccauacc......................   2      1    LT-2
...............................................................................................................................................................................................................uuuccuaccccucccauacc......................   33     0    LT-1
...............................................................................................................................................................................................................uuuccuaccccucccauacc......................   11     0    LCK-3
...............................................................................................................................................................................................................uuuccuCccccucccauacc......................   2      1    LT-3
...............................................................................................................................................................................................................uuuccuaccccucccauacc......................   10     0    JCK-3
...............................................................................................................................................................................................................uuuccuaccccucccauacc......................   12     0    JCK-1
```

```
                                                                                                                                                          Star                                                                                       Mature
ccuuuuuuucaccaaaaucuagacucaauuaauuucucuuuucccuuccuccaccaucugguguauuucuuuucucacuaaaaggauggaagagggaaaggcuuaagagugugggugga agugauagguaugggggguuaggacagagagaaauacucucuuuuucccaauuuauuuauuuuuuaauugggggg aagggaaugaauucuuuccuaccccucccauaccauucauuuccauccuuuccuuc
................................................................................................................................................................................................................uuuccuaccccucccauacc......................  8   0  LT-2
................................................................................................................................................................................................................uuuccuaccccucccauacc......................  18  0  JCK-2
................................................................................................................................................................................................................uuuccuaccccucccauacc......................  28  0  LT-3
................................................................................................................................................................................................................uuuccuaccccucccauacca.....................  1   0  JCK-2
................................................................................................................................................................................................................uuuccuaccccucccauacca.....................  2   0  JCK-3
................................................................................................................................................................................................................uuuccuaccccucccauacca.....................  1   0  LT-3
................................................................................................................................................................................................................uuuccuaccccucccauacca.....................  2   0  LT-1
................................................................................................................................................................................................................uuuccuaccccucccauaccU.....................  1   1  JCK-1
................................................................................................................................................................................................................uuuccuaccccucccauacca.....................  2   0  JT-1
................................................................................................................................................................................................................uuuccuaccccucccauacca.....................  1   0  JCK-1
................................................................................................................................................................................................................uuuccuaccccucccauacca.....................  3   0  LCK-3
................................................................................................................................................................................................................uuuccuaccccucccauaccU.....................  2   1  LT-1
................................................................................................................................................................................................................uuuccuaccccucccauaccU.....................  1   1  JCK-2
................................................................................................................................................................................................................uuuccuaccccucccauaccU.....................  1   1  JCK-3
................................................................................................................................................................................................................uuuccuaccccucccauaccau....................  15  0  JCK-2
................................................................................................................................................................................................................uuuccuaccccucccauaccau....................  6   0  JT-3
................................................................................................................................................................................................................uuuccuaccccucccauaccau....................  5   0  JT-2
................................................................................................................................................................................................................uuuccuaccccucccauaccau....................  4   0  LT-1
................................................................................................................................................................................................................uuuccuaccccucccauaccau....................  7   0  LT-3
................................................................................................................................................................................................................uuuccuaccccucccauaccau....................  10  0  JCK-1
................................................................................................................................................................................................................uuuccuaccccucccauaccUu....................  1   1  LT-1
................................................................................................................................................................................................................uuuccuaccccucccauaccau....................  2   0  LCK-3
................................................................................................................................................................................................................uuuccuacccAucccauaccau....................  1   1  JCK-2
................................................................................................................................................................................................................uNuccuaccccucccauaccau....................  1   1  JCK-2
................................................................................................................................................................................................................uuuccuaccccucccauaccau....................  7   0  JCK-3
................................................................................................................................................................................................................uuuccuaccccucccauaccauu...................  2   0  JCK-3
................................................................................................................................................................................................................uuuccuaccccucccauaccauu...................  3   0  JCK-2
................................................................................................................................................................................................................uuuccuaccccucccauaccUuu...................  2   1  LT-2
................................................................................................................................................................................................................uuuccuaccccucccauaccauu...................  3   0  LT-3
................................................................................................................................................................................................................uuuccuaccccucccauaccauu...................  2   0  LT-2
................................................................................................................................................................................................................uuuccuacccGucccauaccauu...................  1   1  LT-2
................................................................................................................................................................................................................uuuccuaccccNcccauaccauu...................  1   1  JCK-1
................................................................................................................................................................................................................uuuccuaccccucccauaccauu...................  3   0  LT-1
................................................................................................................................................................................................................uuuccuaccccucccauaccauuU..................  1   1  LT-1
................................................................................................................................................................................................................uuuccuaccccucccauaccauuc..................  3   0  LT-1
................................................................................................................................................................................................................uuuccuaccccucccauaccauuc..................  1   0  LCK-3
................................................................................................................................................................................................................uuuccuaccccucccauaccauuc..................  1   0  JCK-1
................................................................................................................................................................................................................uuuccuaccccucccauaccauuc..................  6   0  JCK-3
................................................................................................................................................................................................................uuuccuaccccucccauaccauuc..................  3   0  JCK-2
................................................................................................................................................................................................................uuuccuaccccucccauaccauuc..................  1   0  LT-3
.................................................................................................................................................................................................................uuccuaccccucccauacc......................  6   0  LCK-3
.................................................................................................................................................................................................................uuccuaccccucccauacc......................  3   0  LT-1
.................................................................................................................................................................................................................uuccuaccccucccauacc......................  8   0  JT-1
.................................................................................................................................................................................................................uuccuaccccucccauacc......................  1   0  LT-3
.................................................................................................................................................................................................................uAccuaccccucccauaccauu...................  2   1  JCK-1
.................................................................................................................................................................................................................uuccuaccccucccauaccauu...................  5   0  LCK-3
.................................................................................................................................................................................................................uuccuaccccucccauaccauu...................  2   0  JCK-3
.................................................................................................................................................................................................................uuccuaccccucccauaccauu...................  2   0  LT-1
.................................................................................................................................................................................................................uAccuaccccucccauaccauu...................  2   1  JCK-3
.................................................................................................................................................................................................................uuccuaccccucccauaccauu...................  1   0  JCK-2
.................................................................................................................................................................................................................uuccuaccccucccauaccauuc..................  4   0  JCK-1
.................................................................................................................................................................................................................uuccuaccccucccauaccauuc..................  4   0  JCK-3
.................................................................................................................................................................................................................uuccuaccccucccauaUcauuc..................  1   1  JCK-2
.................................................................................................................................................................................................................uuccuaccccucccauaccauuU..................  1   1  LT-1
.................................................................................................................................................................................................................uuccuaccccucccauaccauuc..................  11  0  JCK-2
..................................................................................................................................................................................................................uccuaccccucccauacc......................  1   0  LT-1
..................................................................................................................................................................................................................uccuaccccucccauacc......................  2   0  LT-3
..................................................................................................................................................................................................................uccuaccccucccauacc......................  2   0  LCK-3
..................................................................................................................................................................................................................uccuaccccucccauacc......................  3   0  JT-2
..................................................................................................................................................................................................................uccuaccccucccauacc......................  1   0  JCK-2
..................................................................................................................................................................................................................uccuaccccucccauacc......................  1   0  JCK-1
..................................................................................................................................................................................................................uccuaccccucccauacc......................  5   0  JT-3
..................................................................................................................................................................................................................uccuaccccucccauacc......................  1   0  LT-2
..................................................................................................................................................................................................................uccuaccccucccauacca.....................  5   0  JT-3
..................................................................................................................................................................................................................uccuaccccucccauacca.....................  5   0  LT-2
..................................................................................................................................................................................................................uccuaccccucccauaccCuu...................  1   1  JCK-1
..................................................................................................................................................................................................................uccuaccccucccauaccauu...................  85  0  JCK-2
..................................................................................................................................................................................................................uccuaccccucccaCaccauu...................  1   1  JCK-2
..................................................................................................................................................................................................................uNcuaccccucccauaccauu...................  1   1  JCK-3
..................................................................................................................................................................................................................uccuaccccucccauaccauu...................  11  0  LT-3
```

```
                                                                                                                                    Star                                                                  Mature
ccuuuuuucaccaaaaucuagacucaauuaauuucucuuucccuuccuccaccaucugguguauuucuuuucucacuaaaaggauggaagagggaaaggcuuaagagugugggguggaagugauagguaugggggguuaggacagagagaaauacucucuuuucccaauuuauuuauuuuuuaauuggggggaagggaaugaauucuuuccuaccccucccauaccauucauuuccauccuuuccuuc
................................................................................................................................................................................................................uccuaccccucccauaccauu...................  20   0  LT-1
................................................................................................................................................................................................................uccuacccAucccauaccauu...................  1    1  JCK-2
................................................................................................................................................................................................................uccuaccccucccauaccauu...................  5    0  LT-2
................................................................................................................................................................................................................uccuaccccucccauaccauu...................  53   0  JCK-3
................................................................................................................................................................................................................uccuaccccucccauaccauu...................  52   0  JCK-1
................................................................................................................................................................................................................uccuaccccucccauCccauuc..................  1    1  JCK-1
................................................................................................................................................................................................................uccuaccccucccCuaccauuc..................  1    1  JCK-2
................................................................................................................................................................................................................uccuaccccucccauaccauuc..................  142  0  JCK-2
................................................................................................................................................................................................................uccuaccccucccauaccauuc..................  23   0  LT-3
................................................................................................................................................................................................................Cccuaccccucccauaccauuc..................  1    1  JCK-2
................................................................................................................................................................................................................uccuaccccucccauaccauuU..................  2    1  LT-3
................................................................................................................................................................................................................uccuCccccucccauaccauuc..................  1    1  JCK-2
................................................................................................................................................................................................................uccuaccccucccauaccauuU..................  3    1  JCK-1
................................................................................................................................................................................................................uccuaccccucccauaccauuU..................  1    1  LT-1
................................................................................................................................................................................................................uccuaccccuAccauaccauuc..................  1    1  JCK-1
................................................................................................................................................................................................................uccuaccccucccauaccauuA..................  1    1  JCK-3
................................................................................................................................................................................................................uccuaccccucccauaccauuU..................  6    1  JCK-2
................................................................................................................................................................................................................uccuaccccucccauaccauuU..................  3    1  JCK-3
................................................................................................................................................................................................................uccuaccccucccauaccauuU..................  3    1  JT-2
................................................................................................................................................................................................................uccuaccccucccauaccauuc..................  16   0  LT-1
................................................................................................................................................................................................................uccuaccccucccauaccauuc..................  8    0  LCK-3
................................................................................................................................................................................................................uccuaccccucccauaccauuU..................  5    1  JT-3
................................................................................................................................................................................................................uccuacccAucccauaccauuc..................  1    1  JCK-3
................................................................................................................................................................................................................uccuaccccucccauaccauuc..................  5    0  LT-2
................................................................................................................................................................................................................uccuaccccucccauaccauuc..................  66   0  JCK-1
................................................................................................................................................................................................................uccuaccccucccauaccauuc..................  101  0  JCK-3
.................................................................................................................................................................................................................ccuaccccucccauacca.....................  2    0  LT-2
.................................................................................................................................................................................................................ccuaccccucccauacca.....................  1    0  LT-3
.................................................................................................................................................................................................................ccuaccccucccauaccau....................  3    0  JCK-3
.................................................................................................................................................................................................................ccuaccccucccauaccauu...................  4    0  LT-2
.................................................................................................................................................................................................................ccuaccccucccauaccauu...................  3    0  LT-1
.................................................................................................................................................................................................................ccuaccccucccauaccauu...................  1    0  LCK-3
.................................................................................................................................................................................................................ccuaccccucccauaccauu...................  3    0  LT-3
.................................................................................................................................................................................................................ccuaccccucccauaccauu...................  12   0  JCK-3
.................................................................................................................................................................................................................ccuaccccucccauaccauu...................  5    0  JCK-1
.................................................................................................................................................................................................................ccuaccccucccauaccauu...................  20   0  JCK-2
.................................................................................................................................................................................................................cUuaccccucccauaccauu...................  1    1  JCK-2
.................................................................................................................................................................................................................ccuaAcccucccauaccauuc..................  1    1  JCK-1
.................................................................................................................................................................................................................ccuaccccucccauaccauuU..................  2    1  JCK-1
.................................................................................................................................................................................................................ccuaccccucccauaccauuc..................  4    0  LT-1
.................................................................................................................................................................................................................ccuaccccucccauaccauuU..................  5    1  JCK-3
.................................................................................................................................................................................................................ccuaccccCcccauaccauuc..................  1    1  JCK-2
.................................................................................................................................................................................................................ccuaccccucccauaccauuc..................  34   0  JCK-2
.................................................................................................................................................................................................................ccuaccccucccauaccauuU..................  5    1  JCK-2
.................................................................................................................................................................................................................ccuaccccucccauaccauuU..................  1    1  LT-3
.................................................................................................................................................................................................................ccuaccccucccauaccauuc..................  5    0  LT-3
.................................................................................................................................................................................................................ccuaccccucccauaccauuc..................  22   0  JCK-1
.................................................................................................................................................................................................................ccuaccccucccauaccauuc..................  32   0  JCK-3
.................................................................................................................................................................................................................ccuaccccucccauaccauucU.................  3    1  LT-2
.................................................................................................................................................................................................................ccuaccccucccauaccauuca.................  1    0  JCK-1
..................................................................................................................................................................................................................cuaccccucccauaccau....................  3    0  LT-1
..................................................................................................................................................................................................................cuaccccucccauaccauu...................  4    0  LCK-3
..................................................................................................................................................................................................................cuaccccucccauaccauu...................  1    0  LT-2
..................................................................................................................................................................................................................Uuaccccucccauaccauu...................  1    1  LT-1
..................................................................................................................................................................................................................cuaccccucccauaccauu...................  5    0  LT-3
..................................................................................................................................................................................................................cuaccccucccauaccauu...................  6    0  LT-1
..................................................................................................................................................................................................................cuaccccucccauaccaCu...................  1    1  LT-2
..................................................................................................................................................................................................................cuaccccucccauaccauu...................  3    0  JCK-2
..................................................................................................................................................................................................................cuaccccucccauaccauuU..................  1    1  LT-2
..................................................................................................................................................................................................................cuaccccucccauaccauuc..................  5    0  JCK-2
..................................................................................................................................................................................................................cuCccccucccauaccauuc..................  1    1  LT-3
..................................................................................................................................................................................................................cuaccccucccauaccauuc..................  6    0  LT-3
..................................................................................................................................................................................................................cuaNcccucccauaccauuc..................  1    1  JCK-1
..................................................................................................................................................................................................................cuaccccucccauaccauuU..................  2    1  JCK-3
..................................................................................................................................................................................................................cuaccccucccauaccauuc..................  3    0  JCK-1
..................................................................................................................................................................................................................cuaccccucccauaccauuc..................  3    0  JCK-3
..................................................................................................................................................................................................................cuaccccucccauaccauuc..................  4    0  LT-1
..................................................................................................................................................................................................................cuaccccucccauaccauuU..................  1    1  LT-1
..................................................................................................................................................................................................................cuaccccucccauaccauuU..................  1    1  JCK-2
..................................................................................................................................................................................................................cuaccccucccauaccauuU..................  3    1  LCK-3
```

Star **Mature**

```
ccuuuuuuucaccaaaaucuagacucaauuaauuucucuuuucccuuccuccaccaucugguguauuucuuuucucacuaaaaggauggaagagggaaaggcuuaagagugugggguggaagugauagguaugggggguuaggacagagagaaauacucucuuuuucccaauuuauuuauuuuuuaauugggggaagggaaugaauucuuuccuaccccucccauaccauucauuuccauccuuuccuuc
...........................................................................................................................................................................................................cuaccccucccauaccauuc..................   5   0   LT-2
...........................................................................................................................................................................................................cuaccccucccauaccauuca.................   2   0   JCK-1
...........................................................................................................................................................................................................cuaccccucccauaccauuca.................   1   0   LT-1
...........................................................................................................................................................................................................cuaccccucccauaccauucU.................   1   1   LCK-3
............................................................................................................................................................................................................uaccccucccauaccauuc..................   1   0   LT-1
....................................................................................................................................................................................................................ccauaccauucauuuccauccu.......   2   0   LT-1
....................................................................................................................................................................................................................ccauaccauucauuuccauccuAu.....   1   1   JCK-3
....................................................................................................................................................................................................................ccauaccauucauuuccauccuAu.....   1   1   JCK-2
.....................................................................................................................................................................................................................cauaccauucauuuccauc.........   1   0   LT-1
.....................................................................................................................................................................................................................cauaccauucauuuccauU.........   1   1   JCK-2
.....................................................................................................................................................................................................................cauaccauucauuuccauccu.......   1   0   LT-3
.....................................................................................................................................................................................................................cauaccauucauuuccauccuu......   2   0   JCK-3
.....................................................................................................................................................................................................................cauaccauucauuuccauccuu......   1   0   JCK-2
.....................................................................................................................................................................................................................cauaccauucauuuccauccuuu.....   2   0   JCK-3
.....................................................................................................................................................................................................................cauaccauucauuuccauccuuu.....   1   0   JCK-2
.....................................................................................................................................................................................................................cauaccauucauuuccauccuuuc....   2   0   JCK-1
......................................................................................................................................................................................................................auaccauucauuuccauc.........   1   0   LT-3
......................................................................................................................................................................................................................auaccauucauuuccauccuu......   1   0   LT-3
......................................................................................................................................................................................................................auaccauucauuuccauccuuu.....   1   0   LT-3
......................................................................................................................................................................................................................auaccauucauuuccauccuuuc....   1   0   LT-1
......................................................................................................................................................................................................................auaccauucauuuccauccuuuU....   1   1   JCK-3
......................................................................................................................................................................................................................auaccauucauuuccauccuuuc....   1   0   LT-2
......................................................................................................................................................................................................................auaccauucauuuccauccuuucc...   3   0   JCK-2
......................................................................................................................................................................................................................auaccauucauuuccCuccuuucc...   1   1   JCK-2
......................................................................................................................................................................................................................auaccauucauuuccauccuuuccuu.   1   0   LT-3
.......................................................................................................................................................................................................................uaccauucauuuccauccuuucc...   2   0   JCK-1
...........................................................................................................................................................................................................................auucauuuccauccuuucc...   2   0   LT-3
...........................................................................................................................................................................................................................auucauuuccauccuuuccu..   2   0   LT-2
...........................................................................................................................................................................................................................auucauuuccauccuuuccuu.   3   0   LT-3
```
